# An All Oxide-Based Imperceptible Thin-Film Transistor with Humidity Sensing Properties

**DOI:** 10.3390/ma10050530

**Published:** 2017-05-13

**Authors:** Kyung Su Kim, Cheol Hyoun Ahn, Won Jun Kang, Sung Woon Cho, Sung Hyeon Jung, Dae Ho Yoon, Hyung Koun Cho

**Affiliations:** Department of Advanced Materials Science and Engineering, SungKyunKwan University, 2006 Seobu-ro, Jangan-gu, Gyeonggi-do, Suwon 16419, Korea; luckykks7@skku.edu (K.S.K.); c2hyoun2@skku.edu (C.H.A.); wjkang@skku.edu (W.J.K.); jumbutter@skku.edu (S.W.C.); wjdtjdgus2@skku.edu (S.H.J.); whhk57@skku.edu (D.H.Y.)

**Keywords:** *a*-IGZO TFT, imperceptible, ITO, humidity sensor

## Abstract

We have examined the effects of oxygen content and thickness in sputtered InSnO (ITO) electrodes, especially for the application of imperceptible amorphous-InGaZnO (*a*-IGZO) thin-film transistors (TFTs) in humidity sensors. The imperceptible *a*-IGZO TFT with 50-nm ITO electrodes deposited at Ar:O_2_ = 29:0.3 exhibited good electrical performances with V_th_ of −0.23 V, SS of 0.34 V/dec, µ_FE_ of 7.86 cm^2^/V∙s, on/off ratio of 8.8 × 10^7^, and has no degradation for bending stress up to a 3.5-mm curvature. The imperceptible oxide TFT sensors showed the highest sensitivity for the low and wide gate bias of −1~2 V under a wide range of relative humidity (40–90%) at drain voltage 1 V, resulting in low power consumption by the sensors. Exposure to water vapor led to a negative shift in the threshold voltage (or current enhancement), and an increase in relative humidity induced continuous threshold voltage shift. In particular, compared to conventional resistor-type sensors, the imperceptible oxide TFT sensors exhibited extremely high sensitivity from a current amplification of >10^3^.

## 1. Introduction

Recently, imperceptible devices that are ultra-thin, ultra-light, and transparent have been spotlighted as next-generation new-concept electronic devices, because they can be fashioned to be undetectable to human touch and sight [1,2,3,4,5]. In particular, sensors with imperceptible characteristics can be applied to various applications such as e-skin devices for checking health signals or monitoring environmental conditions [6,7,8,9,10]. The ultra-light slim devices on polymer substrates can also be attached to objects with round shapes, and transparent sensors can be embedded on commercial glasses such as building windows and tables without blocking visibility.

Typical sensor devices consisting of inorganic semiconductors are of the resistor type, in which the resistance of the semiconductor body increases (or decreases) with increasing oxidation (or reduction) of a gas [11,12,13,14]. More developed semiconductor sensors are based on Schottky or p-n junction diodes, and these exhibited highly responsible and enhanced sensitivity due to their reduced reverse current level. The sensibility performance of semiconductor sensors can be determined by off-state current values; the diode is shown to have very adequate electrical performance. On the contrary, resistor-type sensors comprise nanoparticles or polycrystalline to enhance sensibility by enlarging the reactive surface area [15,16,17,18].

The relatively complex thin-film transistors (TFTs) have been applied as driving devices for display application, rather than sensing devices. These transistors play an intrinsic role of switching or amplifying in solid-state semiconductor devices. The recently developed amorphous-InGaZnO (*a*-IGZO) TFTs show unstable performance against ambient conditions such as light irradiation or water vapor environments [19,20,21], although this drawback as a driving device can be seen as a merit in sensor devices. Nevertheless, research on chemical sensor or humidity sensor applications using oxide TFTs is relatively little due to the complex process and low reproducibility in fabricating such sensors. Our recent studies demonstrated the possibility of low-temperature fabrication of oxide TFTs, and proved that these devices can be fabricated with ultra-slim or transparent characteristics [22].

**Figure 1:**
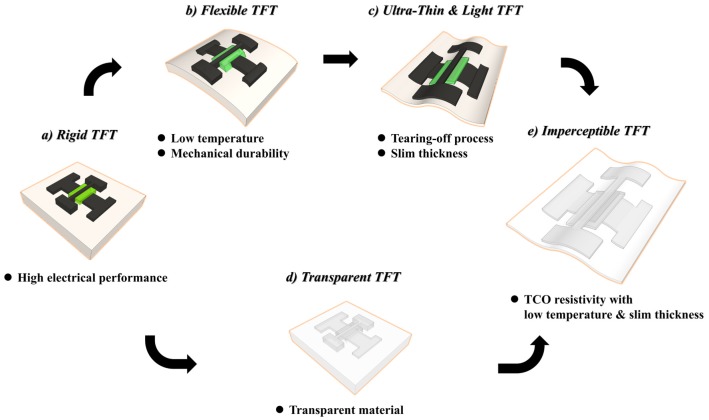
Schematic showing the technical step and core issues for the development of ultra-thin imperceptible thin-film transistors (TFTs): rigid → flexible and transparent → ultra-slim → imperceptible.

To realize imperceptible oxide TFTs with ultra-light and slim physical properties, some technical issues should first be resolved. The first is the development of a low-temperature process for the use of flexible polymer substrates, and the second is the use of fully transparent materials in the channel, electrode, and gate insulators. Finally, the ultra-slim device thickness should be compatible with chemically or mechanically detachable processes. These technical requirements for rigid conventional TFTs are summarized in Figure 1. 

Based on our current technical status, transparent In-Sn-O (ITO) electrodes must be checked for good electric performances, high optical properties, and the possibility of undergoing the wet etching process. In this study, we fabricated high-performance imperceptible TFTs by developing optimized ITO coating conditions at room temperature on a flexible parylene polymer substrate, and conducted humidity sensing tests under different relative humidity conditions.

## 2. Results and Discussion

### 2.1. Resistivity of ITO Via Gas Ratio and Thickness

To fabricate imperceptible TFTs with ultra-light and ultra-slim physical characteristics, all electrodes (source, drain, and gate) must use inorganic-based transparent conductive oxide (TCO) such as ITO for high-speed interconnections. When ITO is used for transparent electrodes, these ITO films should be deposited at low temperatures due to the low glass transition temperatures of flexible polymer substrates. Moreover, the films should be as thin as possible due to their low flexibility. Unfortunately, a low deposition temperature and a very low film thickness significantly enhance the electrical resistivity of the inorganic TCO films [23,24], resulting in the delay and degradation of the electrical signal. In particular, imperfect electrical properties of the TCO layers originate from various defect sites like impurities, vacancies, and anti-site atoms. These defects are very susceptible to any change in the chemical environment, including humidity. Thus, improper TCO coating can induce variations in electrical properties due to humidity change, and hinders the sensing operation in the channel region of the TFT. Previous research has shown that the electrical and optical properties of ITO are strongly dependent on the deposition conditions such as temperature and gas mixture ratio [25]. We changed the Ar:O_2_ gas ratio during the deposition for the optimization of ITO films to be used in imperceptible TFT-based sensors, because the elevation of the growth temperature was limited due to the use of polymer substrate. 

**Figure 2:**
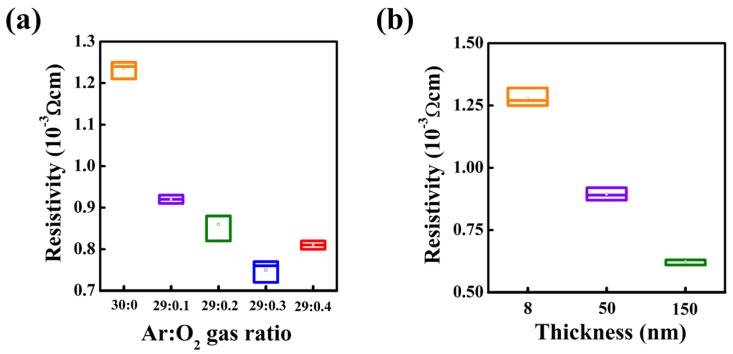
Statistical data on the electrical resistivity of InSnO (ITO) films as a function of (**a**) Ar:O_2_ gas ratio during deposition and (**b**) film thickness.

Figure 2 shows the statistical data on the electrical resistivity of ITO films as a function of Ar:O_2_ gas ratio during deposition and according to film thickness. As shown in Figure 2a, the resistivity of ITO films with similar thicknesses (~100 nm) show a reduction tendency from 1.23 × 10^−3^ Ω∙cm to 0.75 × 10^−3^ Ω∙cm up to an Ar:O_2_ gas ratio of 29:0.3. For the deposition in a pure Ar atmosphere, the oxide films typically exhibited granular grains, less dense pores, and a degradation of transmittance below 80% (not shown here). Because O_2_ gas was injected during the deposition, the pores acting electron scattering were reduced. Therefore, the resistivity of the ITO film decreased. However, at an Ar:O_2_ gas ratio of 29:0.4, the resistivity of ITO started to increase. Excess oxygen flow under room temperature during deposition resulted in semiconducting or insulating electrical properties of the ITO layers, due to the formation of insufficient charge carriers. Thus, the appropriate oxygen content should produce high-quality TCO films with the lowest resistivity (0.75 × 10^−3^ Ω∙cm) at Ar:O_2_ = 29:0.3, with a high transmittance of >90%. Consequently, we fixed the Ar:O_2_ gas ratio at 29:0.3 during ITO deposition by radio frequency (RF) sputtering. Next, we altered the ITO film thickness (147, 50, and 8 nm respectively). In typical oxide films, a decrease in the film thickness is expected to induce frequent electron scattering due to the grain boundary compared to electron transport in thick ITO films [26]. Thus, a thinner film distinctly indicates a larger electrical resistivity. However, considering mechanical durability against bending stress, a decreased ITO thickness is profitable. Under bending stress, the amount of applied stress depended on the distance from a neutral layer that does not receive any bending stress. Thus, the fabrication of thinner TFTs is one of the best ways to delay mechanical breakdown. Figure 2b shows that the decrease in film thickness results in an increase in electrical resistivity, where 150-, 50-, and 8-nm ITO films show resistivity of 0.62, 0.89, and 1.28 × 10^−3^ Ω∙cm. Interestingly, the 50-nm thickness is accompanied by a slight increase of resistivity, and this value is sufficient for the fabrication of imperceptible TFTs. 

### 2.2. Contact Resistivity of ITO/IGZO and Mo/IGZO

The most important parameter for metal electrodes is the contact resistance at the metal-semiconductor junctions. To confirm contact resistivity between the electrode and the oxide channel, the transmission line method (TLM) is a very convenient tool [27]. Many groups have researched the contact resistivity between IGZO films and various electrodes by using TLM [28,29] and other techniques [30,31]. Generally, it is well-known that the contact resistivity between the electrode and the channel contributes on the electrical performance (on current and mobility) of TFTs [32]. Typical electrodes for driving TFT devices in the display application are opaque Mo- and Cu-based electrodes. However, transparency is a prerequisite for the development of imperceptible TFT sensors, and relatively thin TCO electrodes are required for this. Thus, we have compared the contact resistance of thin ITO films deposited at room temperature with that of the popular Mo metal layer on IGZO channels via circular TLM. The contact resistivity can be estimated from the following equation:

(1) $$R_{T}=\frac{R_{S}}{2\pi }\left[\ln\left(\frac{r_{1}}{r_{1}-d}\right)+L_{T}\left(\frac{1}{r_{1}}+\frac{1}{r_{1}-d}\right)\right]$$

where r_1,_ R_T_, R_S_, and L_T_ indicate the radius of the circular pattern, total resistance, sheet resistance, and transfer length traversed by the current flow, respectively. L_T_ is defined as $L_{T}=\sqrt{\left(\rho_{C}/R_{s}\right)}$. d indicates the distances between the circular patterns (10, 15, 20, 25, 30, and 35 μm). 

**Figure 3:**
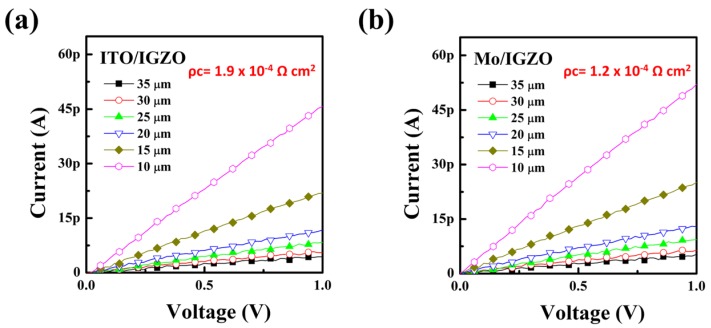
I-V curves obtained from circular transmission line method (TLM) of (**a**) ITO/IGZO and (**b**) Mo/IGZO junctions.

As shown in Figure 3, the estimated contact resistivity at the ITO/IGZO and Mo/IGZO junctions is 1.9 × 10^−4^ Ω∙cm and 1.2 × 10^−4^ Ω∙cm, respectively. These C-TLM results confirm that our ITO films on the IGZO have electrical contact characteristics almost similar to the Mo/IGZO junction, implying the sufficient electrical performance of the thin ITO deposited at room temperature on the IGZO.

### 2.3. Electrical Performance

We fabricated imperceptible ultra-thin TFTs (thickness ≈ 5 μm) using the developed ITO electrodes and evaluated their transfer performances. 

**Figure 4:**
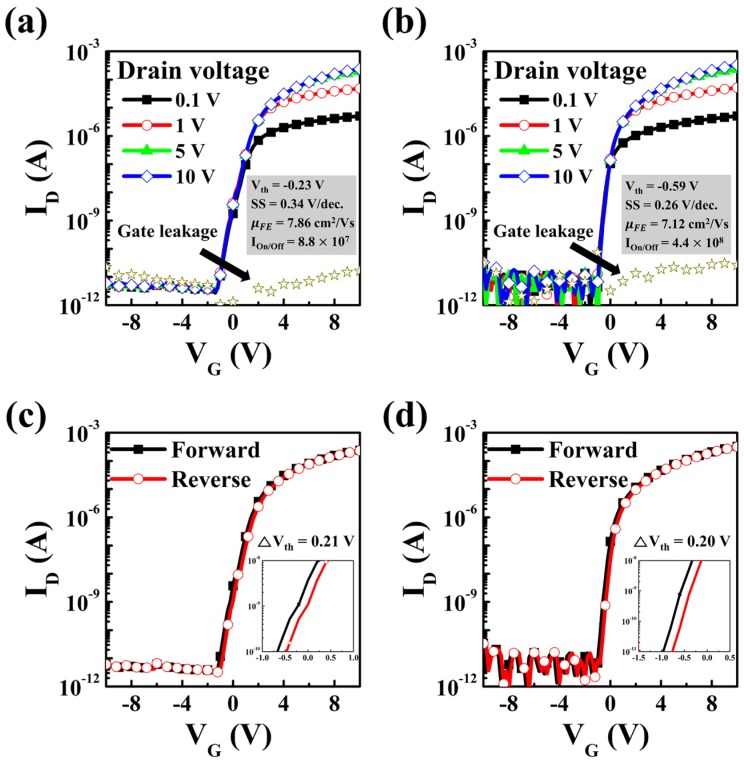
Transfer characteristics (V_D_ = 0.1, 1, 5, and 10 V) and hysteresis curves (V_D_ = 10 V) of imperceptible TFTs with ITO electrodes (**a**,**c**), and ultra-thin TFTs with Mo electrodes (**b**,**d**).

Figure 4a,b show transfer curves at drain voltages of 0.1, 1, 5, and 10 V from all the transparent ultra-thin imperceptible TFTs with transparent ITO electrodes, and ultra-thin TFTs with opaque Mo electrodes on flexible parylene substrates. First of all, µ_FE_, indicating how quickly electrons move through the channel layer, is calculated by the follow equation:

(2) $$\mu_{FE\;}=\;{\left[\frac{L}{WC_{i}V_{D}}\frac{dI_{D}}{dV_{G}}\right]}_{MAX}$$

where L and W are the length and width of channel, respectively, and V_D_, I_D_, and V_G_ are the drain voltage, drain current, and gate voltage, respectively. Sub-threshold swing (SS) values, indicating switching velocity from the off- to the on-state of the TFT, are obtained from:

(3) $$\mathrm{SS}=\;{\left[{\left(\frac{d\log I_{D}}{dV_{G}}\right)}_{MAX}\right]}^{-1}$$

The insets in Figure 4a,b show the summary of representative electrical performances of these TFTs with ITO electrodes and Mo electrodes. Similar to the TLM results, the imperceptible IGZO TFT shows outstanding transfer performances comparable to ultra-slim TFTs based on Mo electrodes, considering the V_th_, µ_FE_, SS and on/off ratio values. The V_th_, µ_FE_, SS and on/off ratio of imperceptible TFT are −0.23 V, 7.86 cm^2^/V·s, 0.34 V/dec and 8.8 × 10^7^. Those of ultra-slim TFT are −0.59 V, 7.12 cm^2^/V·s, 0.26 V/dec and 4.4 × 10^8^. The hysteresis curves in Figure 4c,d show that the imperceptible TFT exhibits marginal V_th_ shift, while the ITO- and Mo-based ultra-slim TFTs exhibit similar ∆V_th_ of 0.21 and 0.20 V. Since the ∆V_th_ in the hysteresis curve depends on the amount of defect sites existing in the channel and gate dielectric interface, we can conclude that there is no meaningful difference in defect density at the channel/gate dielectric interfaces.

### 2.4. Mechanical Stability and Optical Property

Ultimately, the fabricated transparent and ultra-thin/light imperceptible TFT was attached to human skin and arbitrary round objects. 

**Figure 5:**
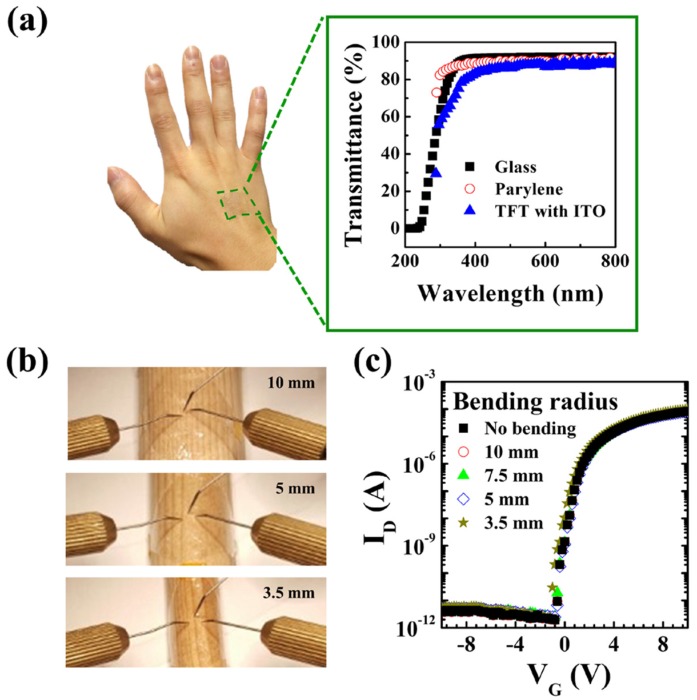
(**a**) Transmittance data of imperceptible TFTs, and reference pure glass and parylene-coated glass. The imperceptible TFT was attached to the skin of a hand and is not clearly recognized; (**b**) Picture showing the bending test of the TFTs attached to wooden sticks with various curvature radii (10, 7.5, 5, 3.5 mm) and (**c**) their transfer characteristics.

Figure 5a shows that we cannot clearly recognize the existence of the TFT on skin due to its high transparency (around 90%) and ultra-slim structure. Transmittances of pure glass and PVA/parylene-coated glass used for comparison are 91.9% and 89.9% at a 550-nm wavelength, and that of our imperceptible TFT is 87.1%. Our imperceptible TFTs are also stably driven without any electrical degradation under bending stress on the round objects, as shown in Figure 5b,c, where the imperceptible TFTs are tested on wooden sticks with curvature radii of 10, 7.5, 5, and 3.5 mm. 

### 2.5. Humidity Sensing Performance

Many researchers have studied the effect of ambient conditions on oxide semiconductors for chemical sensing applications, and their typical performance has been measured in regard to variation of electrical resistance. Unfortunately, resistor-type sensors exhibit relatively low sensibilities (or small changes in the resistance). On the contrary, TFT-based sensor devices with a complex device structure are expected to demonstrate high responsivity due to the low off-current and the intrinsic amplification characteristics of the transistors. According to previous studies, chemical ambience such as gas, pH, and humidity induce the change in important transfer parameters of TFT such as V_th_, SS, and off-current. For display applications, the effect of water vapor on IGZO TFTs has been surveyed; when the IGZO film was exposed to water vapor, an H_2_O molecule or hydroxyl is formed, which donates free electrons to the IGZO channel. These additional electrons increase the electron carrier concentration of the IGZO film and make the film conductive, resulting in an increase in the off-current or negative shift of V_th_ [33]. The high susceptibility of the electrical performance to water vapor suggests the possibility as a sensor device for a humidity test. Noticeably, our transistors are transparent and ultra-slim. Thus, these TFTs can be attached to glass or human skin, and also fabricated on commercial window glasses such as buildings, automobiles, tables, etc. 

**Figure 6:**
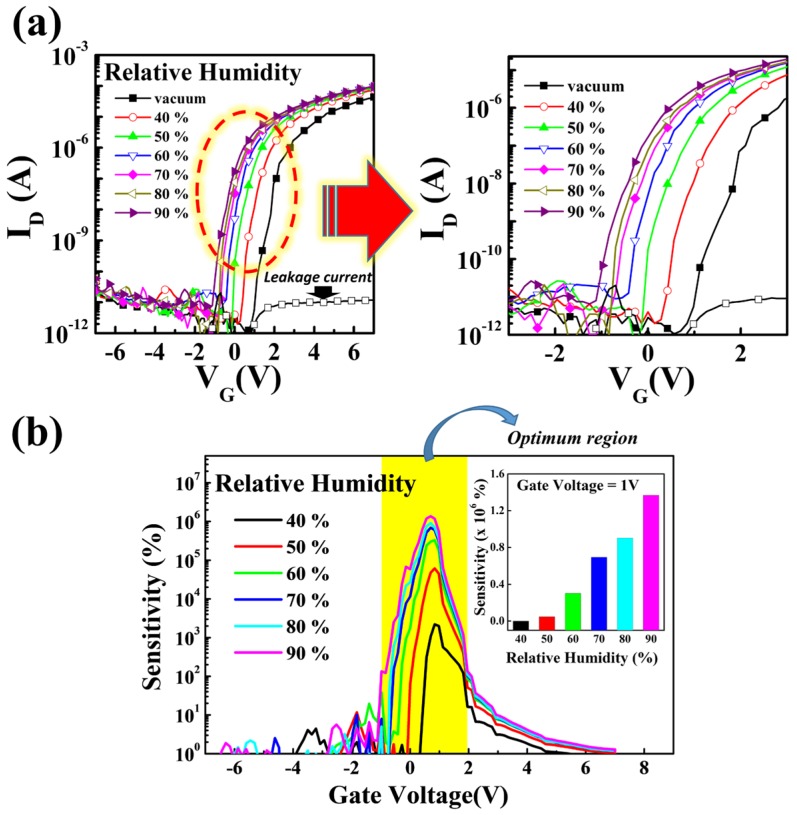
(**a**) Variation in transfer characteristics of imperceptible TFT under various humidity conditions. (**b**) Sensitivity values of imperceptible TFT as a function of gate voltage under different humidity conditions.

Figure 6a shows the variation of electrical performance of the imperceptible IGZO TFT under various humidity conditions (40, 50, 60, 70, 80, and 90%) at V_D_ = 1 V. To investigate the effect of humidity on the TFT performance, we continuously increased the humidity from 40% to 90%, together with a vacuum condition as a reference. When exposed to water vapor, the imperceptible TFT shows a continuous negative shift of V_th_ from 1.55 V (vacuum state) to −0.74 V (90% relative humidity) with negligible change of the off-current level and the SS value. Typically, water vapor can be absorbed on the back channel surface of oxide semiconductors and act as electron donors on the surface. Additional electrons generated from water vapor accelerate the accumulation of the channel layer and cause a negative V_th_ shift. However, no severe variation of the SS value in this device was founded as relative humidity increased. This indicates that the water vapor did not induce an additional charge trapping at channel/gate dielectric interface. To obtain the sensitivity of imperceptible TFTs against relative humidity, sensitivity values can be calculated by:

(4) S= (Ihumidity−Ivacuum)Ivacuum

Unlike the resistor-type sensors, the TFT devices with the third electrode, gate bias, and the applied gate bias exhibit different sensing characteristics. As shown in Figure 6b, the highest sensitivity is observed for V_G_ = −1~2 V at V_D_ = 1 V, implying the low-voltage operation of this sensor. Consequently, we found that high sensing performance can be obtained in the threshold region of the transistors. The insert in Figure 6b shows the calibration curve as a function of the humidity. Our imperceptible TFTs have superior linearity against humidity conditions at a gate voltage of 1V. Many researchers have studied resistive-type humidity sensors based on metal oxide with various structures, such as thin-film [34,35,36], nanoparticles [37], composites [38] and nanocrystals [39]. The sensitivity of resistive-type humidity sensors is mainly below 10^3^. Surprisingly, the sensitivity values of our sensor devices are extremely large (>10^3^). Considering the ultra-light and imperceptible characteristics of the TFT, this sensor is expected to have wide applications.

## 3. Materials and Methods 

### 3.1. Preparation of Substrate

To form ultra-thin substrates, we deposited parylene (~10 μm) polymers via chemical vapor deposition (CVD). To improve the surface roughness of the parylene substrate, we performed UV/ozone treatment for 20 min. For chemical separation between the handling substrate (glass or Si) and the flexible parylene substrate, we additionally coated water-soluble poly vinyl alcohol (PVA, thickness 400 nm) by spin-coating.

### 3.2. Fabrication of TFTs

To investigate the effect of the Ar:O_2_ flow gas ratios on the electrical characteristics of ITO electrodes, we deposited ITO films on glass substrates at various Ar:O_2_ gas ratios (Ar:O_2_ = 30:0, 29:0.1, 29:0.2, 29:0.3, 29:0.4) by radio frequency magnetron sputtering and simultaneously changed the thickness of the ITO films at Ar:O_2_ = 29:0.3. The TFTs for use were fabricated with the bottom gate inverted staggered type structure. The source, drain, and gate electrodes were patterned using negative and positive photolithography (PR) with the wet etchant of HCl:deionized water = 1:5. Next, *a*-IGZO channels were deposited by radiofrequency (RF) magnetron sputtering (150 W, Ar:O_2_ = 28:2, 3 mTorr, 55 nm) and were patterned with negative PR. The channel width and length were 50 and 500 μm, respectively. Then, 40-nm Al_2_O_3_ films with good dielectric property were deposited by atomic layer deposition (ALD) at 150 °C. After the demonstration of the IGZO TFTs, the samples were soaked in deionized water at 70 °C to detach the imperceptible IGZO TFTs from the handling substrates by the dissolution of PVA.

### 3.3. Evaluation of Films and TFTs

The thicknesses of the deposited ITO films were measured by surface profiler equipment and the electrical resistivity of ITO films deposited under various conditions (Ar:O_2_ gas ratio, thickness) were obtained using HMS-3000 hall measurement equipment (Ecopia, Anyang-si, Korea). We used 4145B parameter analyzer (HP, Yokogawa, Japan) to confirm the electrical performance and performance variation of the fabricated TFTs when they were bent with various radii or exposed to different relative humidity levels. Transmittances of imperceptible TFTs were characterized with a UV/visible spectrometer. We characterized sensing performances under different relative humidity conditions in an isolated gas chamber (MS tech). To tune the exact humidity status, we flew air gas passing DI water into the chamber and loaded hygrometer within the chamber. Humidity sensing was performed by checking the transfer performance of imperceptible TFTs in situ with respect to relative humidity.

## 4. Conclusions

We developed oxide TFTs in the order of rigid → separately transparent and flexible → ultra-slim → imperceptible. For the realization of complete imperceptible TFTs with high performance comparable to those on rigid substrates, we surveyed optimized ITO coating conditions with relatively small thicknesses at room temperature. Based on these technical developments, we proposed humidity sensor devices with extremely high responsivity and imperceptible physical characteristics. The ITO electrodes were deposited at an Ar:O_2_ ratio of 29:0.3 by RF sputtering at room temperature and the 50-nm thickness was sufficient for application in the imperceptible TFTs. The imperceptible TFTs exhibited a high mobility of 7.86 cm^2^/V∙s and a stable electrical performance under bending stress. The TFT-based sensors showed the highest sensitivity for the low gate bias of −1~2 V, resulting in low power consumption by this sensor. Compared to conventional resistor-type sensors, the oxide TFT sensors exhibited extremely large sensitivities. Considering that these TFTs are ultra-light and imperceptible, our sensors are expected to show wide applications because they can be patched or embedded on several objects encountered in everyday life.
